# Web-Based Behavioral Intervention Utilizing Narrative Persuasion for HIV Prevention Among Chinese Men Who Have Sex With Men (HeHe Talks Project): Intervention Development

**DOI:** 10.2196/22312

**Published:** 2021-09-16

**Authors:** Meiqi Xin, Neil S Coulson, Crystal Li Jiang, Elizabeth Sillence, Andrew Chidgey, Norman Nok Man Kwan, Winnie W S Mak, William Goggins, Joseph Tak Fai Lau, Phoenix Kit Han Mo

**Affiliations:** 1 Centre for Health Behaviours Research Jockey Club School of Public Health and Primary Care The Chinese University of Hong Kong Hong Kong Hong Kong; 2 School of Medicine University of Nottingham Nottingham United Kingdom; 3 Department of Media and Communication City University of Hong Kong Hong Kong Hong Kong; 4 Department of Psychology Northumbria University Newcastle United Kingdom; 5 AIDS Concern Hong Kong Hong Kong; 6 Health and Care Service Department Hong Kong Red Cross Hong Kong Hong Kong; 7 Department of Psychology The Chinese University of Hong Kong Hong Kong Hong Kong

**Keywords:** narrative persuasion, firsthand experiential stories, online intervention, HIV prevention, sexual behavior, men who have sex with men

## Abstract

**Background:**

In the era of potent antiretroviral therapy, a high level of condomless anal intercourse continues to drive increases in HIV incidence in recent years among men who have sex with men. Effective behavior change strategies for promoting HIV-preventive behaviors are warranted. Narrative persuasion is a novel health communication approach that has demonstrated its persuasive advantages in overcoming resistance to counterattitudinal messages. The efficacy of narrative persuasion in promoting health behavior changes has been well documented, but critical research gaps exist for its application to HIV prevention.

**Objective:**

In this study, we aimed to (1) capitalize on narrative persuasion to design a web-based multisession intervention for reducing condomless anal intercourse among men who have sex with men in Hong Kong (the HeHe Talks Project) by following a systematic development process; and (2) describe the main components of the narrative intervention that potentially determine its persuasiveness.

**Methods:**

Persuasive themes and subtopics related to reducing condomless anal intercourse were initially proposed based on epidemiological evidence. The biographic narrative interview method was used to elicit firsthand experiential stories from a maximum variation sample of local men who have sex with men with diverse backgrounds and experiences related to HIV prevention; different types of role models were established accordingly. Framework analysis was used to aggregate the original quotations from narrators into collective narratives under 6 intervention themes. A dedicated website was finally developed for intervention delivery.

**Results:**

A series of video-based intervention messages in biographic narrative format (firsthand experiential stories shared by men who have sex with men) combined with topic-equivalent argumentative messages were produced and programmed into 6 intervention sessions. The 6-week intervention program can be automatically delivered and monitored online.

**Conclusions:**

We systematically created a web-based HIV prevention intervention derived from peer-generated stories. Strategies used to enhance the efficacy of the narrative intervention have been discussed within basic communication components. This paper describes the methods and experiences of the rigorous development of a narrative communication intervention for HIV prevention, which enables replication of the intervention in the future.

## Introduction

### Men Who Have Sex With Men as an Important Target for HIV Prevention

Although a steadily decreasing trend has been observed for the global HIV burden, the epidemic has continued to expand among men who have sex with men [1]. For example, in Hong Kong, HIV prevalence has increased to 6.54% among men who have sex with men in recent years, while the rate has remained lower than 0.1% in the general population [2]. Despite biomedical advances in antiretroviral therapy and growing access to care and treatment, there is a paradoxical rise in the HIV incidence rate among men who have sex with men that was found to be significantly associated with an increase in condomless anal intercourse [3,4]. The disproportionate risk is also fueled by a relatively low level of HIV testing [5,6]. More efforts are necessary to develop effective behavior change strategies for the promotion of HIV-preventive behaviors among this vulnerable population.

### Gaps in Communicating Persuasive Messages on HIV Prevention

Although health communication is widely acknowledged as an important tool for HIV prevention [7], existing persuasive communications has failed to significantly stimulate behavior change [8]. Previous interventions drew heavily on classic health behavior change theories to improve cognitive determinants (eg, risk perceptions) [7]; however, such interventions are based on the assumption that message recipients are so-called *rational individuals,* and the influence of recipients’ preexisting state in accepting and executing desirable behaviors are omitted [9,10]. Counterattitudinal messages, namely persuasive topics and arguments that differ from recipients’ own values or beliefs [11], are largely used for health behavior promotion. For instance, interventions for HIV prevention are commonly developed to target high-risk groups who engage in unprotected sex or have a low intention to use condoms consistently. Recipients are prone to argue against advocated positions to protect their previously held values or beliefs [11,12], adhering to or even engaging in more of the discouraged behavior [13]. There has long been a need to overcome such resistance to persuasive messages about HIV prevention [14].

### Theoretical Mechanisms Underlying Narrative Persuasion

Over the past decade, narrative persuasion has been recognized as a promising avenue for health communication [12]. A narrative refers to “a representation of connected events and characters that has an identifiable structure [15].” The traditional approach—argumentation—otherwise features expository and didactic communication, with a logical presentation of overt arguments and factual information in the form of reasons and evidence to put forth a claim [12,15,16]. The two communication styles are fundamentally different in terms of information processing. Instead of receiving carefully elaborated explicit persuasive arguments in argumentative communication [17], narrative processing typically involves engagement with narrative elements and identification with characters (ie, vicarious experience of characters’ cognitive and emotional responses) as casually and chronologically related events unfold in a coherent manner [16,18,19].

Narrative persuasion is grounded in both the Extended Elaboration Likelihood Model [18] and the Transportation-Imagery Model [20]; narrative persuasion has the advantage (over argumentation) of inhibiting negative responses to counterattitudinal messages because fewer targets are available for counterarguments, given its structure, with implicit persuasive content embedded in narrative elements; (2) there is less processing capacity and motivation to critically scrutinize core arguments that are presented in narrative format; and (3) identification with narrative characters facilitates psychological distancing from the recipients’ initial story-inconsistent positions [16,18,19,21].

Remarkably, the incorporation of argumentative support further bolsters narrative persuasion [22,23], facilitating cognitive elaboration, rehearsal, and repetition, which leads to an enduring persuasive impact [18,24,25]. Prefacing argumentative messages by narrative messages could potentially predispose recipients to positively process and comprehend the overtly persuasive arguments [26-28]. Recipients could first shift from existing positions temporarily through engagement with the narrative messages, and fully assimilate the advocated positions into their own value structures through positive elaboration of the subsequent argumentation messages [18].

### Harnessing Narrative Persuasion for Behavioral Interventions

Existing empirical evidence supports the efficacy of narrative interventions in promoting health behaviors relative to argumentation or null controls [29,30]. Relatively fewer studies have examined the effect of a combination of narrative and argumentative messages about cancer screening [31-33], diabetes self-care [34], and vaccination [35]; most were found to be more effective in changing the target behavior and its theoretical determinants than a single message format.

Several studies [36-41] have applied narrative communication to HIV prevention, but there are some critical research gaps. First, most narratives took the form of fictional stories (eg, entertainment-education soap opera) that were scripted by researchers; its efficacy in improving HIV-preventive behaviors is controversial [36,37]. However, other narrative types, including experiential stories, have received little exploration. Second, some interventions involved multiple behavior change strategies (eg, condom distribution), and thus, made it difficult to isolate the specific effect of the narrative component [38]. Third, the majority of previous narrative interventions addressed heterosexual risk for HIV [39,40]; we suppose that efficacy of narrative interventions is higher among homosexual people because HIV prevention is more personally relevant to this population group, and personal relevance of a health topic can enhance narrative efficacy [41]. Finally, no studies have been conducted among Asian populations. More research on narrative persuasion in different contexts is warranted because shared stories inherently embody rich cultural information that shapes behaviors and responses to persuasive communication [9,42].

### Utilizing Web-Based HIV Prevention Interventions for Men Who Have Sex With Men

Web-based technology has been recognized as a powerful tool to efficiently reach target populations and deliver behavioral interventions to enhance responses to the ongoing HIV epidemic among men who have sex with men [6]. A recent meta-analysis [43] of 44 studies across 12 countries and regions found that eHealth interventions effectively reduced condomless anal intercourse, reduced multiple sex partnership, and increased the uptake of HIV testing among men who have sex with men. Moreover, a considerable proportion of men who have sex with men actively use the internet for socializing and sex-seeking, which has been found to increase likelihood of condomless anal intercourse, and consequently, risk of HIV infection [44-46]. In Hong Kong, approximately 70% of men who have sex with men engage in sex-seeking online [47]. Hence, web-based interventions can seize the opportunity to promote HIV prevention among men who have sex with men at a crucial point when they tend to engage in risky sexual practices. In addition, Chinese men who have sex with men use the internet as the primary source of information about sexual health, HIV, and other sexually transmitted infections (STIs), and accept the use of eHealth in supporting their sexual health care [48].

In this study, we developed a web-based narrative intervention for HIV prevention among men who have sex with men in Hong Kong (the HeHe Talks Project) to deliver a series of video-based persuasive messages to reduce condomless anal intercourse. Biographic narratives (firsthand experiential stories shared by local men who have sex with men peers) were combined with topic-equivalent argumentative messages.

## Methods

### Ethics and Procedure Overview

**Figure 1:**
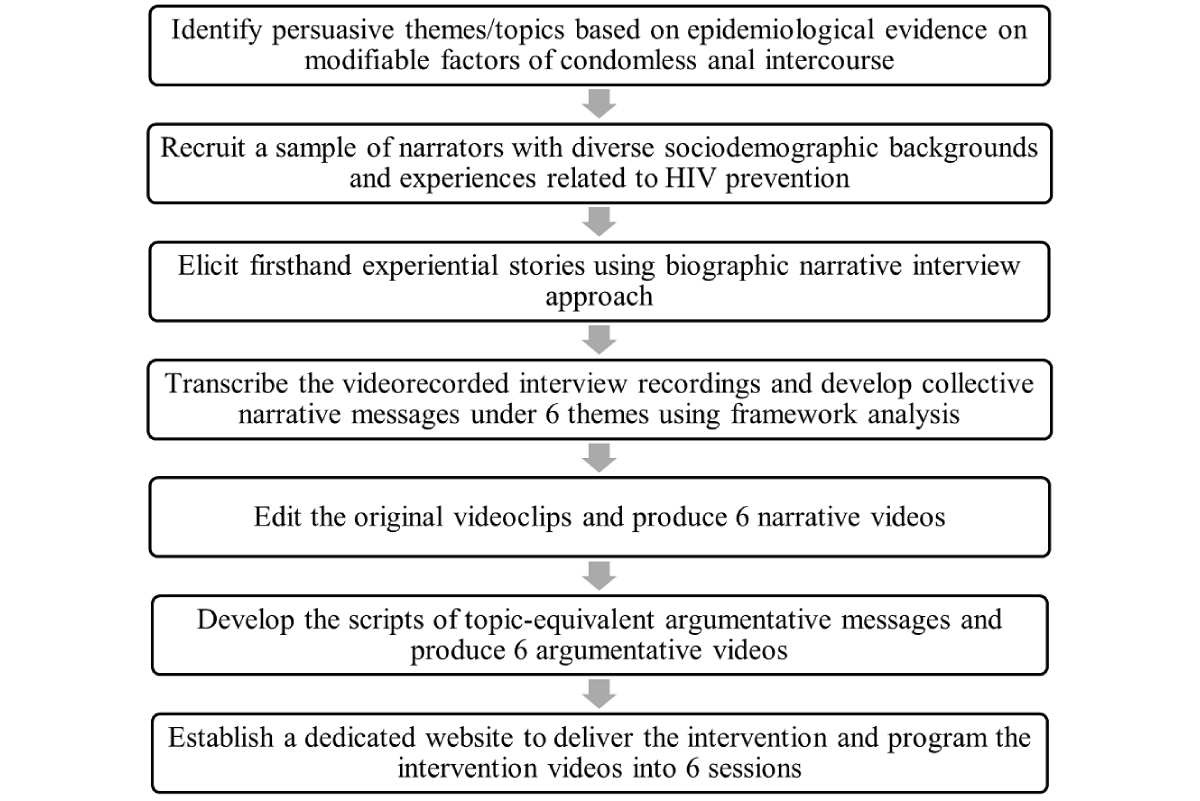
Procedure for developing the narrative intervention program (HeHe Talks).

Informed consent was obtained from all participants involved in this project. Ethical approval was obtained from Joint Chinese University of Hong Kong–New Territories East Cluster Clinical Research Ethics Committee (reference number 2014.274-T). A roadmap of the development procedure is presented in Figure 1.

### Identifying Persuasive Themes and Subtopics

We conducted a review of modifiable and significant predictors of HIV/STI infection and condomless anal intercourse among local men who have sex with men in publications and government documents to inform topic selection. Factors were categorized into cognitive (eg, risk perceptions), interpersonal (eg, sex partnership), contextual (eg, substance use), and sexual practice–specific (eg, group sex). In addition, it has been suggested that HIV testing behavior should be addressed simultaneously, given its significant relationship with condomless anal intercourse among Chinese men who have sex with men [49,50]. Six session themes were initially proposed: knowledge about HIV/STI, condom use and partnership, condom use and risky contexts, condom use and substance use, efficacy of condom use, and HIV testing (Multimedia Appendix 1).

Qualitative evidence was obtained by conducting a focus group discussion with 5 Hong Kong men who have sex with men (age: range 28-38 years). The focus group was led by a public health researcher and a health psychologist and was used to assess knowledge, views, and information needs regarding HIV prevention and condomless anal intercourse. Most interviewees had university-level educations and above (4/5) and had full-time jobs (4/5); 2 interviewees reported consistent condom use for anal sex, and 1 had engaged in substance use–facilitated anal sex in the previous 6 months.

This focus group informed refinement of the intervention topics. For example, the participants emphasized the phenomenon of “complacency about HIV” among local men who have sex with men (ie, that strong belief about antiretroviral therapy efficacy contributed to low-risk perceptions and high motivation to engage in condomless anal intercourse). Proposed intervention topics were sent to several HIV specialists; we further revised the topics based on their feedback. For example, they suggested focusing on drug use other than alcohol consumption to address reducing substance use–facilitated condomless anal intercourse; accordingly, the subtopic *impact of alcohol use on condomless anal intercourse* was removed.

### Recruiting Narrators and Operationalizing the Role Models

The Sabido entertainment-education strategy [25] was applied to establish 3 types of role models: (1) positive characters, who engage in consistent condom use while having anal intercourse and undergo regular HIV testing, and consequently, maintain sexual health; (2) negative characters, who engage in condomless anal intercourse and do not regularly undergo HIV testing, and are negatively affected (eg, infected with HIV or STI); and (3) transitional characters, who once engaged in risky sexual behaviors and suffered negative consequences and then adopted more positive attitudes and behaviors. Role modeling has been adopted in previous entertainment-education programs for HIV prevention [37,39]. Maximum variation sampling was used to recruit 2 to 3 HIV-positive and 6 to 7 HIV-negative narrators with different experiences and backgrounds [51].

Criteria for all narrators were (1) men who have sex with men; (2) age ≥18 years; (3) Hong Kong residents; (4) able to speak Cantonese fluently; and (5) of sufficient mental and physical capability to take part in a 2-hour interview. Criteria for HIV-positive narrators were (1) confirmed or high probability of having acquired HIV by sexual transmission; (2) encountered negative experiences due to living with HIV; (3) once or currently undergoing antiretroviral treatment; and (4) having engaged in any condomless anal intercourse with male sex partners prior to the diagnosis. Criteria for HIV-negative narrators were (1) having undergone at least 1 HIV test in the previous year, with the most recent test having yielded a negative result; (2) consistently used condoms with male sex partners (in every sexual encounter in the previous year); and (3) having positive perceptions about condom use for HIV prevention. Prospective narrators were nominated and approached by the research team and a collaborating nongovernmental organization (AIDS Concern). A survey was administered to establish their eligibility and to create a profile for each individual, with background information (eg, age and sexual partnership) and specific experiences related to HIV prevention (eg, contracting HIV/STI infections, engaging in condomless anal intercourse).

### Developing Persuasive Messages in Narrative and Argumentation Formats

Biographic-Narrative Interpretive Method [52,53] was employed to elicit narratives for the development of health promotion messages. A semistructured interview guide was drafted around the proposed topics and tailored to each narrator’s profile. Two interviewers (MX and NK) and a professional camera operator were involved in the individual interviews with each of the selected narrators. One of the interviewers (NK) was a senior practitioner from the collaborating nongovernmental organization who had rich experience in providing community support services to local men who have sex with men; he took a leading role in the narrative interviews and preinterview negotiations, particularly with HIV-positive narrators, to ensure that the questions were delivered in a comfortable and respectful manner.

Interviewers first briefed the narrator on interview procedures and established rapport. Individual interviews started with a single question that was thematically or temporally focused, to elicit narratives of personal experiences, such as life after being diagnosed as HIV positive or an occasion of safe sex practices at a gay venue. Narrators were free to tell their story in the manner of their choosing (to provoke the narration within their own system of relevancy), and interviewers merely provided nondirectional facilitative support. Keyword notes were also taken to document the topics of interest arising in the original narrative, which helped inform follow-up questions for additional narrative to enrich the overarching story. After narrative-seeking questions, interviewers asked additional questions about topics that had not been raised or for purposes other than narrative extraction (eg, to make a persuasive appeal to the audience).

Videorecordings of the interviews were transcribed. Framework analysis was conducted to develop themed narrative messages using NVivo (version 11, QSR International) in 5 steps [54]: (1) familiarizing: carefully reading the transcripts and observational notes to gain a comprehensive understanding of the data; (2) framing: devising and refining a coding framework drawn on both predefined and emergent themes and subtopics; (3) indexing: systemically sorting original narrative quotations within the thematic framework; (4) charting: grouping indexed narratives from different narrators for each coded subtopic; (5) mapping: aggregating subtopics and corresponding quotations in a coherent manner under each of the 6 session themes to generate collective narrative messages. For each subtopic, argumentative messages were drafted based on the narrative content, by presenting didactic statements, factual claims, logical reasons, and statistical evidence.

### Producing Intervention Videos and a Web-Based Delivery Platform

The videorecordings of the original narratives were segmented and re-organized to create 6 videos for the themes derived from the framework analysis. A background profile was presented for each narrator at first appearance in the videos; persuasive appeals from 1 or 2 narrators were used as epilogues. Videos were sent to the narrators to get their approval. Three male speakers (a nongovernmental organization staff and 2 research assistants) were invited to deliver scripted, videorecorded argumentative messages for each theme. Supplementary material, such as openings, subtitles, images, and background music, were added, and the finalized videos were programmed into 6 intervention sessions. Each included 2 videos with narrative (15-20 minutes) and argumentative (8-10 minutes) messages and a postvideo quiz set to enhance participants’ dedication to intervention content. The narrative videos were longer than the topic-equivalent argumentative videos due to the inherent natures of storytelling (ie, portraying a series of events) and argumentation (ie, listing facts and evidence). A dedicated website was developed to deliver intervention sessions automatically, and a content management system was established for real-time monitoring of engagement in the program.

## Results

### Narrator Characteristics

A total of 36 men who have sex with men were screened, and 9 men were selected as narrators (Table 1). Two men, who had been diagnosed as HIV-positive 3 years prior, served as negative role-model narrators. The other men had recent HIV-negative test results (within 1 year): 3 men had never engaged in condomless anal intercourse with a male sex partner in their lifetime, and thus, served as positive role-model narrators; 4 men had once engaged in condomless anal intercourse but had consistently used condoms in the past year, and thus, served as transitional role-model narrators.

Narrators had varying sexual practices (Table 2): 3 had regular partners, and 5 had casual partners; 4 had engaged in both insertive (taking a top role) and receptive (taking a bottom role) anal intercourse; 6 had engaged in *casual fun*—anal intercourse with casual sex partners in collective sex environments (eg, gay saunas, group sex events [55]); 5 had engaged in *chem-sex*—anal intercourse while under the influence of psychotropic substances (eg, methamphetamine).

**Table 1:** Background characteristics of the 9 narrators.

Narrator (role model type)	Age (years)	Employment	Years as member^a^	HIV^b^ status	History of other STIs^c^ (type)	Type of HIV testing venues visited
A (negative)	24	Unemployed	Not disclosed	Positive	Yes (syphilis)	Community organization
B (negative)	24	Unemployed	>10	Positive	No	Public social hygiene clinic
C (transitional)	30	Full-time	>10	Negative	Yes (urethritis, pubic lice)	Public social hygiene clinic; community organization
D (positive)	38	Full-time	>10	Negative	No	Community organization; private clinic
E (transitional)	26	Full-time	>5	Negative	No	Public social hygiene clinic; community organization
F (transitional)	36	Full-time	>10	Negative	Yes (syphilis)	Community organizations
G (positive)	30	Full-time; Part-time	>10	Negative	No	Public social hygiene clinic; community organization
H (transitional)	32	Full-time	>10	Negative	No	Community organization
I (positive)	22	Student	>5	Negative	No	Community organization

^a^Period between self-identification as homosexual or bisexual and involvement in the local community of men who have sex with men.

^b^HIV: human immunodeficiency virus.

^c^STIs: sexually transmitted infections.

**Table 2:** Characteristics of the 9 narrators related to sexual practices (ie, anal intercourse with male sex partners prior to diagnosis or in the past year).

Narrator (role model type)	Sexual partnership	Sexual role	Consistent condom use^a^	Casual fun^b^ (venue)	Chem-sex^c^ (drug type^d^)
A (negative)	Not recorded	Not recorded	No	No	No
B (negative)	Not recorded	Not recorded	No	Yes (gay sauna; private party)	Yes (meth, g water, foxy, rush poppers)
C (transitional)	Regular; casual	Top; bottom	Yes (recent)	Yes (gay sauna; private party)	Yes (rush poppers)
D (positive)	Regular	Top	Yes (lifetime)	No	No
E (transitional)	Casual	Top; bottom	Yes (recent)	Yes (gay sauna)	Yes (meth, g water, foxy)
F (transitional)	Casual	Bottom	Yes (recent)	Yes (gay sauna)	Yes (meth, foxy, ecstasy)
G (positive)	Regular	Top	Yes (lifetime)	No	Yes (rush poppers)
H (transitional)	Regular; casual	Top; bottom	Yes (recent)	Yes (gay sauna)	No
I (positive)	Casual	Top; bottom	Yes (lifetime)	Yes (gay sauna)	No

^a^The response *recent* referred to having once engaged in any condomless anal intercourse in lifetime but consistently using condoms within the past year; the response *lifetime* referred to having never engaged in condomless anal intercourse in lifetime.

^b^This refers to engaging in anal intercourse with casual sex partners at any local public venues.

^c^This refers to taking illicit drugs during or prior to anal intercourse.

^d^Meth, g water, foxy (ie, foxy methoxy), rush poppers, and ecstasy as slang for methamphetamine; gamma hydroxybutyrate/gamma butyrolactone; 5-methoxy-N,N-diisopropyltryptamine; inhalant alkyl nitrites; and 3,4-methylenedioxy-methamphetamine, respectively, were reported.

### Topics and Contents of Persuasive Messages

There are 6 intervention sessions in total (Table 3); all sessions start with persuasive messages in the narrative format. The first session (A New Era of HIV Prevention) introduces the outcomes of HIV infection in the era of potent antiretroviral therapy. The HIV-positive peers narrate their experiences of receiving a diagnosis and living with HIV, with a focus on the severity of infections, negative influences on physical health and personal life, and co-infections with other STIs. The second session (Partnership and Protected Sex) includes experiences of condom use with different types of sexual partners, including actively negotiating condom use and making sexual decisions, resisting sexual pressure from partners, and balancing emotional relationships and safe sex practices. The third session (Casual Fun and Protected Sex) presents both obstacles to condom use experienced in high-risk contexts (eg, dark rooms in gay saunas and group sex parties) and practical skills for coping with unintentional condomless anal intercourse. The fourth session (Chem-Sex) focuses on potential exposure to psychotropic substances at local gay venues, negative influences of substance use on sexual practices and physical health, and problems related to drug addiction. The fifth session (Efficacy of Condom Use) focuses on consistent and correct condom use for anal intercourse as well as its efficacy in preventing STIs. The last session (HIV Testing) includes experiences of undergoing HIV testing and counseling at different service sites and emphasizes the importance of regular testing while adhering to safe sex practices. Each narrative video ends with narrators appealing to the listeners. For example, the first session includes a positive message from a HIV-positive narrator; he talks about gradually accepting and recognizing his infection status, adhering to treatment, and having hope for a new future.

**Table 3:** An overview of topics in the narrative messages for each intervention session.

Themes and subtopics of narrative messages			Message sources (narrator and role model type)
**Session 1: A New Era of HIV Prevention**			
	1. HIV diagnosis	A (negative) and B (negative)	
	2. Life impact of living with HIV	A (negative) and B (negative)	
	3. Problems encountered during HIV treatment	A (negative) and B (negative)	
	4. Co-infection with other sexually transmitted diseases	A (negative)	
**Session 2: Partnership and Protected Sex**			
	1. Active communication about condom use and assessment of partner(s)’ HIV risk	D (positive), C (transitional), and E (transitional)	
	2. Assertive responses to unwanted condomless sex	A (negative), D (positive), G (positive), C (transitional), and E (transitional)	
	3. Barriers to condom use when in an emotional relationship	D (positive), G (positive), C (transitional), and E (transitional)	
**Session 3: Casual Fun and Protected Sex**			
	1. Risk of condomless sex with casual partners and norm about condom use at gay saunas	B (negative), D (positive), I (positive), C (transitional), E (transitional), and F (transitional)	
	2. Unintentional condomless sex encountered during casual fun	B (negative), D, I (positive), C (transitional), E (transitional), and F (transitional)	
	3. Contextual risk of group sex parties	B (negative), D (positive), C (transitional), and H (transitional)	
**Session 4: Chem-Sex**			
	1. Impact of drug abuse on sexual practices	B (negative), E (transitional), F (transitional), and H (transitional)	
	2. Impact of drug abuse on other health outcomes	B (negative), D (positive), C (transitional), E (transitional), and F (transitional)	
	3. Exposure to drugs when seeking sex	B (negative), D (positive), G (positive), I (positive), C (transitional), E (transitional), and F (transitional)	
	4. Drug addiction	B (negative), E (transitional), and F (transitional)	
**Session 5: Efficacy of Condom Use**			
	1. Protective efficacy of condom use	D (positive), G (positive), I (positive), and E (transitional)	
	2. Correct condom use	D (positive), I (positive), C (transitional), and E (transitional)	
	3. Consistent condom use	D (positive), G (positive), C (transitional), F (transitional), and G (transitional)	
**Session 6: HIV Testing**			
	1. Local HIV testing services	D (positive), G (positive), C (transitional), E (transitional), and H (transitional)	
	2. Regular HIV testing	A (negative), G (positive), I (positive), C (transitional), and H (transitional)	

The topic-equivalent argumentative messages are delivered after narratives. For example, for the subtopic *Consistent condom use* (in Session 5), the narrative presents experiences of condomless sex and STI infections from an HIV-positive narrator, and the argumentative message is

If the sexual partner is HIV-positive, one could get infected through only a “single” episode of unprotected sexual encounter, ...research studies demonstrated that there was no significant difference in HIV risk between men who have sex with men who “inconsistently use condoms” and those who “have never ever used condoms.”

After videos, a quiz question is asked. For example, “According to the video messages, what type of immune cell was primarily attacked by HIV?” One correct response options (“CD4 cell”) and 2 false response options (“CD3 cell” and “CD8 cell”) are provided. Feedback and reinforcement are not given after the quiz, although users can return to the video during and after the quiz.

### Web-Based Intervention Program

The program website can deliver the intervention automatically in a standardized manner: the 6 intervention sessions can be released sequentially on a weekly basis, and each session is accessible upon completion of the preceding session. Users can be assigned a unique password-protected account to access intervention content within a 6-week period, and activities are automatically recorded (eg, the dates and time of completing an intervention video). The program can also send out reminders when a new session is released, or users fail to engage in any activity for 2 to 3 weeks.

## Discussion

### General

We developed a theory-based and evidence-based narrative communication intervention for HIV prevention among men who have sex with men. The communication strategies used by this intervention program to enhance the potential narrative efficacy can be systematically discussed within the 4 basic components of health communication: message, source, recipient, and channel [12].

### Message

Narratives took the form of firsthand experiential stories, in contrast to commonly used fictional stories [12]. To maintain the authenticity of personal experience narratives, which is the key to exploiting its advantages (ie, being perceived as credible, evoking high engagement and inhibiting counterarguing) over those of professionally generated stories in persuasion [15,56], we used well-developed methods for biographic narrative interviews to obtain stories [52]. The narrators were always given the space and time to narrate without any interruption; while the interviewers performed active listening by intermittently giving prompts, with nonverbal posture or empathetic and unobtrusive mirroring of the emotions expressed, throughout the storytelling. In addition, the intervention messages were produced by directly extracting biographic accounts from the videorecorded interview. Narratives conveyed in first-person are more effective than third-person narratives for STI prevention [35], because an individual is more likely to mentally embody characters’ perspectives (ie, identification with characters) [57]. These strategies also strengthened the incorporation of cultural codes including visual presentations, language, and idioms that could heighten narrative engagement [58]. Furthermore, this narrative intervention featured subsequent provision of argumentative support. The argumentative messages were developed to match the themes and subtopics of the narrative messages, to reinforce the predisposition created by narrative persuasion but to avoid provoking counterargument [18].

### Source

Collective narratives were extracted from a maximum variation sample of local men who have sex with men peers in this study to improve the comprehensiveness of persuasive content [15] and appeal to different audience segments [25]. The diversity of narrators allowed 3 types of characters to be established. The transitional role model was utilized the most in order to reduce selective avoidance of counterattitudinal messages about HIV prevention [19] and increase perceived homophily and realism of the characters, and thus, facilitate narrative processing [18], and enhance self-efficacy in changing risky sexual behaviors [59]. Positive and negative role models were also used to reinforce the consistency of condom use. Moreover, biographic narratives were a representation of the narrators’ views of their own life-course, beliefs, interpretative schemata, and principles of judgement [52], which provided authentic and comprehensive portrayals of the characters, and in particular, avoided fostering negatively biased images of HIV-positive peers. For instance, the narrators with a history of HIV/STI shared their experiences of infection and risky sexual encounters as well as how they strived to adhere to treatments and improve their HIV-preventive behaviors.

### Recipients

The targeted recipients are Chinese men who have sex with men, a highly marginalized community where members are strongly connected to each other [60]; social norms and community involvement have been found to be positively associated with their HIV-preventive behaviors [61,62]. To produce culturally authentic messages and engage the recipients [9], members of the local men who have sex with men community were actively involved throughout the intervention development, including determining persuasive topics, screening eligible narrators, assisting in narrative interviews, and generating and delivering intervention messages. Such participatory strategies can be applicable to other similar contexts where the community tend to share motives and values regarding health behavior.

### Channel

The intervention comprises web-based videos. Video-based narratives have demonstrated strengths in building affection, facilitating identification with characters, and message elaboration, thereby promoting behavior changes [29,63,64]. Narrators’ ability to organize and articulate stories in videorecorded interviews were carefully evaluated during the recruitment. The web-based delivery platform allows navigation of the content at the user’s own pace without the presence of researchers; users are less likely to mask emotional responses and experience greater immersion when they view narratives alone [56].

### Conclusions

In response to the call for innovative health communication approaches for HIV prevention, this paper describes the scientific rationale and rigorous procedure of applying narrative persuasion to promote HIV-preventive behaviors among men who have sex with men. This web-based narrative communication intervention can be easily replicated in other contexts and can be incorporated into comprehensive HIV prevention services.

## Appendix

Multimedia Appendix 1 A summary of factors of HIV/STI infection and related behaviors among men who have sex with men in Hong Kong.

## Abbreviations

HIV: human immunodeficiency virus
STI: sexually transmitted infections
