# Supplementary material for: Web-Based Behavioral Intervention Utilizing Narrative Persuasion for HIV Prevention Among Chinese Men Who Have Sex With Men (HeHe Talks Project): Intervention Development
Source: J Med Internet Res. 2021 Sep 16;23(9):e22312. doi: 10.2196/22312 (PMC8485190; doi:10.2196/22312)
Supplement: Multimedia Appendix 1 [file jmir_v23i9e22312_app1.docx]

Multimedia appendix 1. A summary of factors of HIV/STI infection and related behaviours among MSM in Hong Kong.

| **Study** | **Sample** | **Outcome** | **Associated factors [category]** | **Proposed intervention themes** |
| --- | --- | --- | --- | --- |
| 1. Hong Kong Department of Health 2012 [1]; Wong 2013 [2]  The PRiSM survey | N=994  90.0% Chinese; median age: 30 years | Positive HIV/STI status | 1. having anal sex within the last 6 months [sexual practice-specific factors] ^*^  2. more than 3 male sex partners [interpersonal factors]  3. engaged in group sex in the last 6 months [contextual & sexual practice-specific factors]  4. concurrent use of recreational drugs during anal sex [contextual factors]  5. received money/rewards for anal sex [contextual factors]^*^  6. having regular sex partner only [interpersonal factors]  7. consistent condom use with non-regular sex partner within last 6 months [interpersonal factors] | condom use and partnership;  condom use and risky contexts;  condom use and substance use |
| 2. Hong Kong Department of Health 2015 [3]  The HARiS survey | N=1026  98.6% Chinese; 77.6% aged 20-39 years | HIV-positive status | 1. involved in group sex [contextual & sexual practice-specific factors]  2. consumption of alcohol and drugs before and during sex in the preceding 6 months [contextual factors] | condom use and risky contexts;  condom use and substance use |
| 3. Leung 2015 [4] | N=311  over 90.0% Chinese; median age: 25 years | 1. Condomless anal intercourse with regular male sex partners  2. Casual sex partnership | 1. visited only a single gay venue (versus frequenting multiple venues) [interpersonal & contextual factors]  2. sex seeking exclusively via Internet (versus via venues) [interpersonal factors] | condom use and partnership;  condom use and risky contexts |
| 4. Cai 2014 [5] | N=285  53.6% aged 20-30 years | Condomless anal intercourse with regular male sex partners | 1. substances use prior to having anal  sex [contextual factors]  2. worry that condom use symbolizes mistrust [cognitive & interpersonal factors]  3. perceived partner’s acceptance of condom use [cognitive & interpersonal factors]  4. sexual impulsivity [sexual practice-specific factors] | condom use and partnership;  condom use and risky contexts;  condom use and substance use;  efficacy of condom use |
| 5. Lau 2016 [6] | N=213  77.0% aged ≤35 years | Condomless anal intercourse with regular male sex partners | 1. participant’s suggestion to have condomless anal intercourse [cognitive & interpersonal factors]  2. discussion about condom use prior to sex [interpersonal factors]  3. partner’s suggestion to have condomless anal intercourse [interpersonal factors]  4. perception that partner would like to use a condom [cognitive & interpersonal factors]  5. planning to use a condom [cognitive factors]  6. sex took place overseas^*^, during a weekday^*^ and at recreational venues [contextual factors]  7. availability of condoms (placed at the sex venue; partner possessed a condom) [interpersonal & contextual factors]  8. alcohol use [contextual factors] | condom use and partnership;  condom use and risky contexts;  condom use and substance use |
| 6. Li 2014 [7] | N=215  74.0% aged ≤35 years | Condomless anal intercourse with non-regular male sex partners | 1. at least three previous anal sex experiences with the partner [interpersonal factors]  2. perceived himself and the partnership having asymmetrical sexual experience [cognitive & interpersonal factors]  3. perceived the partner being feminine/masculine; liking toward the partner [interpersonal factors]  4. having discussed condom use [interpersonal factors]  5. perception that the partner liked to use condom [cognitive & interpersonal factors]  6. participant’s suggestion to use condom [interpersonal factors]  7. partner’s suggestion to use condom [cognitive & interpersonal factors]  8. planning to use a condom [cognitive factors]  9. availability of condoms (placed at the sex venue; partner possessed a condom) [interpersonal & contextual factors]  10. display of condom use promotion materials at the venue [contextual factors] ^*^ | condom use and partnership;  condom use and risky contexts;  condom use and substance use;  efficacy of condom use |
| 7. Lau 2014 [8] | N=433  87.8% aged 18-34 years | condomless anal intercourse | disclosure of sexual orientation to family members [cognitive & interpersonal factors] ^*^ | NA |
| 8. Tsui 2010  [9] | N=566  72.6% aged 18-29 years | condomless anal intercourse | 1. Best friends supporting their sexual orientation [contextual factors] ^*^  2. perceived discrimination against  MSM [cognitive & contextual factors] ^*^ | NA |
| 9. Wong 2004 [10] | N=187  mean age: 8.16 years | condomless anal intercourse | 1. feelings toward condom use [sexual practice-specific factors]  2. perceived vulnerability to STD/AIDS infections [cognitive factors]  3. self-acceptance of homosexual sexual orientation [cognitive factors]  4. disclosure of homosexual sexual orientation and related favorable attitudes [cognitive & interpersonal factors] ^*^  5. perceived discrimination due to homosexual sexual orientation [cognitive & contextual factors] ^*^  6. involved with local gay communities [contextual factors] ^*^ | knowledge about HIV/STI;  condom use and partnership;  condom use and risky contexts;  condom use and substance use;  efficacy of condom use |
| 10. Gu 2011 [11] | N=577  58% aged ≥25 years | HIV testing | 1. attitudes, subjective norms, perceived control and behavioural intention regarding testing [cognitive factors]  2. Perceived fear of contracting HIV [cognitive factors]  3. perceived discrimination towards local MSM [cognitive & contextual factors] ^*^ | knowledge about HIV/STI;  HIV testing |
| 11. Lau 2013 [12] | N=245  52.7% aged ≤25 years | HIV testing (intention) | 1. perceived necessity to participate in HIV test regularly [cognitive factors]  2. perceived descriptive norms about HIV test [cognitive factors]  3. perceived higher chance of having sex with people living with HIV [cognitive factors]  4. perceived higher chance of having unprotected anal intercourse in the next six months [cognitive factors] | knowledge about HIV/STI;  efficacy of condom use;  HIV testing |
| 12. Wang 2017 [13] | N=144  62.4% aged 18-30 | HIV testing | 1. perceived benefit of HIV testing (early detection and better treatment outcomes, protect partners and increase trust, and relief) [cognitive factors]  2. perceived psychological barriers of HIV testing (stigmatization by service providers, embarrassment, and privacy) [cognitive factors]  3. perceived self-efficacy [cognitive factors] | knowledge about HIV/STI;  HIV testing |

*These factors were not used to inform the development of intervention messages as they were not modifiable in a behavioural intervention or not the intervention targets (e.g., abstinence).

References

1. Special Preventive Programme. FACTSHEET on PRiSM - HIV Prevalence and Risk behavioural Survey of Men who have sex with men in Hong Kong 2011 Hong Kong: Hong Kong Department of Health; 2012 [Available from: <http://www.info.gov.hk/aids/english/surveillance/sur_report/prism2011e.pdf>.

2. Wong HTH, Wong KH, Lee SS, Leung RWM, Lee KCK. Community-Based Surveys for Determining the Prevalence of HIV, Chlamydia, and Gonorrhoea in Men Having Sex with Men in Hong Kong. Journal of Sexually Transmitted Diseases. 2013;2013:8. doi: 10.1155/2013/958967.

3. HARiS - HIV and AIDS Response Indicator Survey 2014 for Men who have Sex with Men Hong Kong: Hong Kong Department of Health; 2015 [Available from: <http://www.chp.gov.hk/files/pdf/oth_rep2015_msm_e.pdf>.

4. Leung KK, Poon CM, Lee SS. A Comparative Analysis of Behaviors and Sexual Affiliation Networks among Men Who Have Sex With Men in Hong Kong. Archives of Sexual Behavior. 2015;44(7):2067-76. doi: 10.1007/s10508-014-0390-3.

5. Cai Y, Lau JTF. Multi-dimensional factors associated with unprotected anal intercourse with regular partners among Chinese men who have sex with men in Hong Kong: a respondent-driven sampling survey. Bmc Infect Dis. 2014;14(1):205. doi: 10.1186/1471-2334-14-205.

6. Lau JTF, Mo PKH, Gu J, Hao C, Lai CHY. Association of Situational and Environmental Factors With Last Episode of Unprotected Anal Intercourse Among MSM in Hong Kong: A Case-Crossover Analysis. AIDS Education and Prevention. 2016;28(1):26-42. doi: 10.1521/aeap.2016.28.1.26.

7. Li J, Lau JTF, Gu J, Hao C, Lai CHY. Event-Specific Risk Factors Predicting Episodes of Unprotected Anal Intercourse with Male Nonregular Partners among Men Who Have Sex with Men Using Case-Crossover Study Design. BioMed Research International. 2014;2014:15. doi: 10.1155/2014/475195.

8. Lau JTF, Feng TJ, Liu XL, Gu J, Tsui HY, Hong FC, et al. Associations between Cognitive, Sociocontextual, and Affective Variables and Unprotected Anal Intercourse among Men Who Have Sex with Men-A Comparative Study Conducted in Two Chinese Cities. BioMed Research International. 2014;2014:9. doi: 10.1155/2014/970975.

9. Tsui HY, Lau JTF. Comparison of risk behaviors and socio-cultural profile of men who have sex with men survey respondents recruited via venues and the internet. BMC Public Health. 2010;10(1):232. doi: 10.1186/1471-2458-10-232.

10. Wong CY, Tang CSK. Sexual Practices and Psychosocial Correlates of Current Condom Use Among Chinese Gay Men in Hong Kong. Archives of Sexual Behavior. 2004;33(2):159-67. doi: 10.1023/B:ASEB.0000014330.67201.1b.

11. Gu J, Lau JTF, Tsui H. Psychological factors in association with uptake of voluntary counselling and testing for HIV among men who have sex with men in Hong Kong. Public Health. 2011;125(5):275-82. doi: 10.1016/j.puhe.2011.01.010.

12. Lau JT, Gu J, Tsui HY, Wang Z. Prevalence and associated factors of intention to participate in HIV voluntary counseling and testing for the first time among men who have sex with men in Hong Kong, China. Preventive medicine. 2013;57(6):813-8. doi: 10.1016/j.ypmed.2013.09.005.

13. Wang Z, Lau JTF, She R, Ip M, Jiang H, Ho SPY, et al. Behavioral intention to take up different types of HIV testing among men who have sex with men who were never-testers in Hong Kong. AIDS Care. 2018;30(1):95-102. doi: 10.1080/09540121.2017.1338659.
